# Supplementary material for: Are carbon emissions trading and green financial instruments synergistic? -Comprehensive quantitative research based on content analysis
Source: PLoS One. 2024 Mar 7;19(3):e0298601. doi: 10.1371/journal.pone.0298601 (PMC10919601; doi:10.1371/journal.pone.0298601)
Supplement: S1 Data — (ZIP) [file pone.0298601.s001.zip › Data/地级市Z_F工作报告.zip!/╣π╢1⁄2/╤⌠╜¡╩╨/└┤╫╘╣1⁄2╓┌║┼í╛┬φ┐╦╩2╛▌═°í┐_2004.docx]

各位代表：

　　我代表市人民政府向大会作政府工作报告，请予审议，并请政协委员和其他列席人员提出意见。

　　　　　　　　　　　　　　过去五年工作回顾

　　本届政府任期从1999年3月开始，是跨世纪的五年。五年来，在中共广东省委、省人民政府和中共阳江市委的正确领导下，在市人民代表大会及其常务委员会和市政协的监督支持下，市政府高举邓小平理论伟大旗帜，全面贯彻“三个代表”重要思想，以建设现代化海滨城市为总目标，以发展为第一要务，以结构调整为主线，以体制创新和科技创新为动力，积极实施“追赶型、科教兴市、可持续发展”三大战略，认真抓好“六个做”（做大工业，做优农业，做强海洋产业，做旺旅游业，做好招商引资，做美环境），打好“三张牌”（民营牌、海洋牌、旅游牌），全力推进工业化、城镇化和农业产业化。国民经济实现快速健康发展，社会各项事业全面进步，精神文明建设和民主法制建设取得丰硕成果。市三届人大一次会议确定的各项任务全面完成。

　　——经济实力跃上新台阶

　　2003年全市生产总值231.6亿元，比上年增长12.6%，比1998年增长72.3%，五年年均增长11.5%。人均生产总值8930元，首次突破1000美元，比1998年增长60.3%，年均增长9.9%。生产总值在全省21个地级以上市的排位由1998年的第18位上升到第16位，人均生产总值由1998年的第16位上升到第13位。财政收入增幅较大， 2003年地方财政一般预算收入6.6亿元，按同口径计算，是1998年的2.2倍，全市可支配财力14.9亿元，财政收支保持平衡。金融形势稳定，全市金融机构人民币各项存款余额192.7亿元，比1998年增长68.5%，贷款余额107.2亿元，剔除剥离贷款后比1998年增长2.3%。经济结构调整取得显著成效。农业结构进一步优化，特色农业加快发展，农业产业化经营稳步推进，农业基础地位不断增强，2003年全市农业总产值118亿元，比上年增长6.4%，五年年均增长6.8%。1999年至2001年连续3年获得省粮食责任制考核先进市称号。工业发展环境不断改善，经济效益综合指数不断提高，2003年全市工业总产值226亿元，比上年增长18%，五年年均增长14.9%。第三产业稳步发展，2003年第三产业增加值74.4亿元，比上年增长13.9%，五年年均增长13.6%。三次产业增加值比重由1998年的41.5：29.9：28.6转变为33：34.9：32.1。城市化水平从第四次人口普查的17%提高到第五次人口普查的41.9%。

　　——区域特色经济活力增强

　　民营经济成为国民经济的重要组成部分。实施一系列优惠扶持政策，为民营企业发展营造良好环境，促进了民营经济的大发展、大提高。到去年底，全市民营企业3.8万家，其中民营工业企业8268家，分别比1998年增加3600家和1827家。1999年以来，民营经济以年均20%以上的速度增长，民营经济对国民经济增长的贡献率由1998年的53.6%提高到去年的69.3%。2003年民营工业产值占全市工业总产值的77.9%，培育了喜之郎果冻、美景牌冷却塔、YC深沟球轴承、旺通饼干、十八子菜刀、银鹰剪刀、永光剪刀等一批省名牌产品和驰（著）名商标，中国菜刀中心、中国剪刀中心、中国小刀中心先后落户阳江。2001年，阳江被授予“中国刀剪之都”称号。

　　海洋渔业持续发展。大力实施《关于加快发展海洋渔业的决定》，促进海洋渔业稳步发展，建成了全国最大的吊桩牡蛎和泥蚶养殖基地、广东省重要对虾生产基地和加工出口基地。加强渔港基础设施建设，闸坡渔港成为我省唯一的国家级中心渔港。举办南海（阳江）开渔节，打造海洋渔业品牌。1999年以来，全市水产品总量和人均占有量连续5年居全省第一。2003年全市水产品总量86.4万吨，比1998年增长18.9%，年均增长3.1%；渔业总产值42.4亿元，比1998年增长37.1%，年均增长6.5%，其中出口总额1.2亿美元，比1998年增长9倍，年均增长58.8%。

　　旅游品牌初步形成。坚持把旅游业作为支柱产业，制定《关于加快旅游业发展的决定》，全面修订旅游发展总体规划，整合“山海湖泉林洞”资源，建设海陵岛大角湾和春湾凌霄岩为重点的五大景区，建成了合山、春都两个温泉度假区，开展了创建中国优秀旅游城市和全市十大景区评选活动，成立了市旅游协会，加强对旅游行业的管理和服务。从2000年起每年举办旅游文化节，把旅游、文化、体育、会展、商贸结合起来，提高阳江旅游知名度和影响力。2001年我市被评为“中国优秀旅游城市”，海陵岛大角湾风景区荣获国家AAAA级滨海旅游风景区、省级科技旅游示范区和省文明景区称号。2003年阳春凌霄岩被评为国家地质公园。去年全市接待过夜游客234.9万人次，旅游总收入13.5亿元，分别比1998年增长51.5%和71.7%，年均增长8.7%和11.4%。

　　——各项改革不断深化

　　按照中央和省的部署，积极推进市、县、镇三级政府机构改革，市政府行政编制精简32%。认真做好撤并镇工作，减少了9个镇，增加4个街道办事处，调整面为18.8%。开展行政审批制度改革，取消行政审批事项363项，清理和规范市政府各部门的审批事项，政府职能逐步向经济调节、市场监管、社会管理和公共服务方面转变。推行政务公开。对经营性土地使用权实行公开招标拍卖，对国家投资建设工程、政府采购实行公开招投标，有形建筑、土地、人才、劳动力等要素市场加快发展，市场配置资源的作用不断增强。国有企业改革稳步推进。采取多种形式放开搞活国有中小企业，基本实现了国有企业三年改革与脱困目标。进一步完善国有资产管理、营运和监督体系，组建了2家市国有资产经营公司，并对10家国有企业实行资产授权经营。积极推进社会保障体系建设。到去年底，全市企业养老、失业参保人数分别比1998年增长72.49%和171.9%。企业离退休人员养老金发放全部实行社会化。稳步推进城镇职工基本医疗保险制度、医疗机构和药品生产流通体制改革。理顺了农村电力管理体制，积极推进城乡“同网同价”，农村到户电价降到0.79元/千瓦时。全面开展农村税费改革，全市农民人均减负114.2元，减负率为81.1%。社会各项改革取得新进展，社会主义市场经济体制不断完善。

　　——外经贸工作取得新突破

　　抓住我国加入世贸组织的新机遇，优化出口商品结构，支持民营生产型企业自营进出口，扶持中小企业开拓国际市场，推动对外贸易持续发展。2003年，全市进出口总额8.1亿美元，比上年增长28.8%，其中出口总额6.9亿美元，比上年增长38.5%，五年年均增长4.4%。认真实施“外向带动”战略，进一步优化投资环境，降低营商成本，增强对外商的吸引力，利用外资工作取得新成绩。2003年，全市实际吸收外商直接投资1.3亿美元，比上年增长31%；五年累计实际吸收外商直接投资4.5亿美元，年均增长17%。

　　——城乡市场有效拓展

　　积极采取“政府搭台、企业唱戏”的办法，开展各种经贸活动，开拓国内市场，“阳货”的国内市场占有率得到巩固和提高。市内市场繁荣，居民消费结构逐步升级，房地产、汽车、信息、教育等消费市场进一步扩大。大力整治涉农收费，切实减轻农民负担，拓展农村消费市场。进一步抓好各类市场建设，积极培育和健全商品流通网络，促进了城乡市场销售的稳步增长。目前全市共有各类市场144个，市场经营面积41万平方米。2003年，社会消费品零售总额108.1亿元，集贸市场成交额48.7亿元，分别比1998年增长75.3%和34.7%，年均增长11.9%和6.2%。

　　——基础设施和城市建设成效显著

　　全市五年累计完成全社会固定资产投资225亿元，年均增长13.3%，其中2003年完成固定资产投资57亿元，比上年增长20.1%。交通建设取得较好成绩，建成了广东西部沿海高速公路阳江段、开阳高速公路阳江段、阳阳铁路、省道S113线阳春北段等重点项目。全市公路通车里程4419公里，比1998年增加1222公里，提前实现省到市通高速公路、市到县通一级公路的目标。全市39个镇基本开通了二级以上公路，行政村实现了通机动车。能源建设发展较快，新建、扩建了春城220KV和桂山、平冈110KV输变电工程和农城网工程，推进了阳江核电站、阳西火力发电厂、阳江抽水蓄能电站、海陵岛风力发电场等重大项目的前期工作。水利建设不断加强，开展了“三年大会战”，完成了162宗小Ⅰ、小Ⅱ型水库达标建设和25.6公里堤围除险加固任务，启动了城乡水利防灾减灾工程建设。城市面貌有了很大改观，新建和改造了市区10多条起点较高的道路和街道，新建了市体育馆、污水处理厂、汽车总站、中国刀具城会展中心、市人民医院住院大楼、市中心血站、青少年宫等一批市政工程和教育、文化、卫生设施，改造了人民广场、南国风筝场和一批商业广场、购物步行街、风貌街等商业设施，新建了景湖花园等10多个高起点的住宅小区，推进了市文化广场、图书馆、科技馆、博物馆、群众艺术馆和世纪公园的建设。完善了城市管理体制，城市综合执法和专项整治进一步加强。市区绿化、美化、净化、亮化工作成效显著，鸳鸯湖广场被评为广东省城市十大光亮工程优秀景点。目前，市区建成区面积扩大到33平方公里，市区常住人口增加到近30万人。

　　——科技教育事业发展加快

　　制定了《关于依靠科技进步推动产业结构优化升级的决定》，加大科技投入，大力实施科技兴市10项工程和科技创新9项计划，抓好10家重点工业企业工程技术研究开发中心建设，其中4家被批准组建省级工程技术研究开发中心。设立省级民营科技园，加快科技成果转化和推广。全市实施各类科技项目381项，其中国家级3项，省级72项，获省市科技进步奖167项。全市共有省级高新技术企业22家，比1998年增加19家；省级民营科技企业107家。专利工作保持粤西地区领先地位， 5年共申请1716件，实施率达95%以上。科技进步对工业经济增长的贡献率不断提高，比1998年提高5.6个百分点达到45%。阳江高新技术产业开发区建设取得初步成效。目前已开发土地2.3平方公里，在建工业企业43家，其中7家已建成投产。教育事业迈出新步伐。坚持教育优先发展，增加教育投入，深化教育改革，推进素质教育。“普九”教育成果进一步巩固提高，高中阶段教育加快发展，成人教育、职业教育和高等教育稳步推进，兴办了阳江职业技术学院。确立了“以县为主”的教育管理体制。合理调整中小学布局，撤并112所中小学校分教点，改扩建121所中小学，易地新建15所中小学，改造山区、老区小学122所，有10所学校晋升为省一级学校。 2003年，全市小学适龄儿童入学率为99.9%，初中适龄人口入学率为98%，初中毕业生升学率为58.9%。教育质量明显提高，2003年全市共有8373人进入专科以上高等院校，录取率达81.4%，比上年提高16.9个百分点。

　　——城乡人民生活水平明显提高

　　2003年农村居民人均纯收入4100元，城镇在岗职工人均工资收入10718元，五年年均增长4.7%和7.4%。居民住房条件进一步改善，全市城镇居民人均住房面积32.1平方米，比1998年增加10.3平方米。认真落实“两个确保”和“三条保障线”工作，离退休人员养老金和国有企业下岗职工基本生活费按时足额发放。积极推进“十项民心工程”，每年办好12件实事，努力帮助困难群众解决住房难、饮水难、就医难、打官司难、子女入学难等问题。切实抓好就业和再就业工作，大力推进市劳动力中心市场和技工学校建设，认真落实再就业各项政策，帮助下岗工人特别是“4050”人员再就业，城镇登记失业率控制在3.5%以下。重视资源环境保护和生态建设，市区绿化覆盖率为29.3%，全市森林覆盖率为52.6%，自然生态保护区覆盖率为10.6%，城乡居民生活环境明显改善。

　　——民主法制建设进一步加强

　　坚持市政府的重大事项决策前提交市委常委会讨论。坚持和完善向人大及其常委会报告工作、向政协和各民主党派通报情况的制度，自觉接受监督。认真办理人大代表议案、建议和政协委员提案。发挥民主党派、工商联、无党派人士参政议政作用和工会、共青团、妇联组织的桥梁纽带作用。城乡基层组织建设和民主法制建设不断加强，村务公开、厂务公开扎实推进。加强政府法制工作和执法监督，落实行政执法责任制和执法过错追究制，规范行政执法主体及其行为，依法行政水平逐步提高。认真抓好党风廉政建设和领导干部的廉洁自律工作，开展行风评议活动，从源头上预防和治理腐败。坚决纠正损害群众利益的不正之风。积极开展普法教育、法律服务。审计、监察等工作更好地为经济社会发展服务。加强整顿和规范市场经济秩序工作，加大物价监管力度。信用建设迈出新步伐，市场环境有效改善。高度重视安全生产，狠抓薄弱环节和基础性工作，安全生产形势保持稳定。切实抓好反走私工作。深入开展严打整治斗争，加强社会治安综合治理，严厉打击各种刑事犯罪和“法轮功”等邪教组织的破坏活动，开展“禁毒03工程”，扫除“黄赌毒”等社会丑恶现象。开通了市长热线电话，做好群众来信来访工作，化解人民内部矛盾，社会保持稳定。

　　——精神文明建设和社会各项事业全面进步

　　深入开展学习邓小平理论、“三个代表”重要思想和党的十六大精神活动。全面实施《公民道德建设实施纲要》，积极开展公民道德建设活动。广泛开展群众性精神文明创建活动，公民素质和城市文明程度进一步提高，去年荣获“广东省文明城市”称号。以建设文化名城为目标，发展文化经济和文化产业，推进文化与经济融合。广东海上丝绸之路博物馆筹建和“南海1号”沉船打捞工作取得了阶段性成效。新闻出版、广播电视事业不断发展，群众文化娱乐活动丰富多彩，文艺创作日趋活跃，文化市场健康发展。2003年举办了“中华诗词现象”研讨会，阳江被授予“诗词之市”称号。医疗卫生事业明显进步。重视农村卫生工作，发展社区卫生服务，建立完善卫生监督执法和疾病预防控制体系。加强城乡爱国卫生、医疗服务、疾病控制和药品监督工作。2002年，我市被授予“广东省卫生城市”和“广东省灭蝇先进城区”称号。在抗击“非典”斗争中，政府果断决策，靠前指挥，周密部署，及早采取措施，取得了“非典”零病例的重大胜利。无偿献血工作连续两次获得全国先进城市称号。积极开展中国儿童慈善一日捐活动，荣获“中国公益明星城市”称号。成功举办了第二届市运会，承办了九运会沙滩排球赛、男子室内排球赛和第一届亚洲沙滩排球锦标赛等国际和全国性大型体育赛事。切实抓好人口与计划生育工作，人口自然增长率由1998年的9.06‰降至2003年的6.68‰，先后4次获得省政府的年度表彰。殡葬改革工作由后进变先进，2000年以来连续4年受到省政府的表彰。国防教育、双拥共建、人民武装和民兵预备役工作取得新成绩，征兵工作连续16年获全优单位，连续三次被评为“全省双拥模范城”，2003年被评为“全国双拥模范城”。民政、外事、侨务、民族、宗教、妇女儿童、人防、气象、地震、档案、市志等各项工作都有新发展。

　　各位代表，过去五年我市国民经济和社会发展取得的成就，是省委、省政府和市委正确领导的结果，是各级人民代表大会及其常务委员会、人民政协和各民主党派监督支持的结果，是全市人民团结奋斗、努力拼搏的结果。在此，我代表市人民政府向全市广大工人、农民、知识分子、干部职工、驻阳江人民解放军、武警部队官兵和各界人士表示崇高的敬意！向长期关心支持我市建设的香港特别行政区同胞、澳门特别行政区同胞、台湾同胞、海外侨胞表示衷心的感谢！

　　过去五年，我市国民经济快速发展，经济增长质量明显提高，综合经济实力显著增强，人民群众得到了更多实惠，成绩来之不易。我们的主要体会是：第一，必须高举邓小平理论伟大旗帜，全面贯彻“三个代表”要求，解放思想，实事求是，与时俱进，开拓进取，把中央和省的要求与阳江实际紧密结合起来，创造性地开展工作；第二，必须把发展作为第一要务，狠抓经济建设不放松，坚持发展不动摇，抢抓机遇，发挥优势，扬长补短，加快发展区域特色经济；第三，必须充分调动全市干部群众的积极性和创造性，加强团结，凝聚民心，焕发活力，努力营造心齐实干的良好氛围；第四，必须以体制创新和科技创新为动力，不断完善社会主义市场经济体制，推动经济结构和产业结构的优化升级，努力提高经济综合竞争力；第五，必须扩大对内对外开放，以开放促改革、促发展，充分利用民资民力，积极吸引外资外力，大力发展开放型、混合型经济，不断增强经济发展的活力和后劲；第六，必须坚持依法行政，认真履行政府经济调节、市场监管、社会管理和公共服务职能，着力营造优质高效、依法透明的政务环境；第七，必须树立和落实科学发展观，坚持以人为本，统筹发展，正确处理改革、发展、稳定的关系，正确处理经济建设与人口、资源、环境保护的关系，推动经济社会全面、协调、可持续发展。

　　在肯定成绩的同时，我们也清醒地认识到存在的困难和问题仍然不少。主要是：经济结构不合理，产业竞争力不强，经济总量不大；城市化水平较低，城镇规划、建设和管理水平有待提高；投资软硬环境有待进一步优化，招商引资工作亟需加大力度；高素质人才缺少，劳动就业结构性矛盾突出；财政收支压力较大，城乡建设和社会事业欠账较多；城镇居民和农村群众收入增幅不大，部分群众生活还比较困难；教学水平和质量仍需进一步提高；整治市场经济秩序和社会治安力度有待加强；精神文明建设和民主法制建设还存在不少薄弱环节。面对这些问题和困难，我们必须高度重视，进一步采取有力措施，逐步加以解决。

　　　　　　　　　　　今后五年奋斗目标和2004年工作安排

　　同志们，新世纪的头20年，是我国重要的发展机遇期，党中央、国务院已对全面建设小康社会的伟大事业作出全面部署，省委、省政府就我省如何加快发展、率先发展、全面、协调、可持续发展，继续在全国当好排头兵作出了一系列战略决策和工作部署，张德江书记、黄华华省长视察阳江时对我们提出了殷切的期望，260万阳江人民期待着阳江早日摘掉“欠发达”的帽子，早日过上宽裕的小康生活。在这个发展的关键时刻，我们一定要抓住机遇，励精图治，加快发展，迎头赶上。今后五年，是我市贯彻落实党的十六大和十六届三中全会精神，全面建设小康社会、基本实现社会主义现代化的重要时期。我市经济社会要实现跨越式发展，要在2020年同全省一起同步基本实现社会主义现代化，就必须紧紧抓住发展这个第一要务，充分把握我省实施区域协调发展战略、加快东西两翼经济发展的有利时机，加强同“大珠三角”间的经贸合作与交流，不断探索和实施加快发展、跨越发展的战略新思路、新途径，实现全面、协调、可持续发展。

　　今后五年政府工作总的指导思想是：以邓小平理论和“三个代表”重要思想为指导，全面贯彻中共十六大和十六届三中全会精神，按照省委、省政府的工作要求和市第四次党代会的战略部署，以全面建设小康社会为总目标统揽全局，树立和落实科学发展观，以发展为第一要务，以工业化为主攻方向，以增强综合实力和城市竞争力为核心，积极实施跨越式、科教兴市、可持续发展三大战略，坚持抓好“六个做”、打好“三张牌”，发展临海工业，壮大区域特色经济和县域经济，建设连接“大珠三角”区域交通体系，打造广东电力能源基地，把阳江建成工业新城、生态绿城、旅游美城、文化名城。

　　今后五年的奋斗目标和总体要求：全市生产总值突破400亿元，年均增长12%以上，综合竞争力跃上新台阶，为2020年与全省同步进入全面小康社会，基本实现社会主义现代化奠定坚实的基础。我们要在历届市委、市政府打下的工作基础上，求发展规律之真，务经济发展之实，努力做到“八个新突破”：一是发展思路要有新突破。树立和落实科学发展观，坚持以人为本，实现人的全面发展；坚持统筹城乡发展，统筹山区与沿海的区域发展，统筹民营经济、特色经济、海洋经济与招商引资，统筹人与自然和谐发展，统筹社会经济协调发展；坚持跨越式、科教兴市、可持续发展三大战略并举，重在壮大实力，焕发活力，提高竞争力；坚持政府引导、市场主导、企业自主发展的统一，以完善的体制、机制保障，推动经济社会全面、协调、可持续发展。二是解放思想要有新突破。坚持用马列主义、毛泽东思想、邓小平理论和“三个代表”重要思想统领各项工作、指导实践，克服小富即安思想，树立求大发展意识；克服粗放经营思想，树立质量效益意识；克服“等、靠、要”思想，树立开拓进取意识；克服无所作为思想，树立抢抓机遇意识；克服自我封闭思想，树立开放兼容意识；克服贪图享乐思想，树立艰苦奋斗意识，更新思想观念，与时俱进，创造性地开展工作。三是政府作风建设要有新突破。树立科学发展观和正确的政绩观，坚持执政为民，着力坚持战略思维与工作落实的统一，推动经济建设中重大工程项目的落实，解决经济社会发展中的突出矛盾，着力解决关系人民群众切身利益的突出问题，努力建设“为民、务实、廉洁、高效”政府。四是重大项目建设要有新突破。认真做好重大项目的规划储备工作，各县（市、区）每年筛选1-2个大项目作为储备项目，争取纳入省的笼子。市重点抓好10万吨大型码头、高新区临港工业城、30万吨特殊钢项目，推动阳江港至云浮高速公路和合山、平冈两个220KV输变电工程等重大项目的规划建设，力促“四大发电”项目早立项、早建设、早投产。五是招商引资工作要有新突破。建立招商引资工作新机制，逐级签订责任书，落实责任制，建立奖惩制度，形成党政齐抓，干群参与的浓厚氛围，以基础设施建设为根本，以园区为载体，以机制创新为动力，以干部的作为为桥梁，以招商洽谈文化活动为契机，力促招商引资取得大的突破。六是县域经济发展要有新突破。以农民增收、工业增效、财政增长、后劲增强为目标，加快县域经济发展。各县（市、区）要在大力发展工业的基础上，结合实际，突出重点。阳春市要以凌霄岩获得国家地质公园为契机，全力发展旅游业、中药材加工及山区特色农业；阳东县要重点推动工业园区建设，积极发展生态旅游农业；阳西县要大力发展海洋养殖业和品牌农业；江城区要加快发展现代流通业和服务业；海陵岛试验区要加快发展滨海旅游业；高新区要积极利用外资发展临海工业。七是第三产业发展要有新突破。强化优势，突出特色，整合资源，壮大旅游业，活跃商贸物流业，发展现代服务业，拓展房地产业，培育文化产业，提高第三产业在国民经济中的比重。八是城市建设要有新突破。以提升城市综合竞争力为主攻方向，在完善市区建成区配套建设，规范管理的同时，大力推进沿东门南路、二环南路向南至沿海高速公路联络线5平方公里 “城南新区”的建设，努力把它建设成为一个体现生态型人居理念、体现岭南水乡特色的现代化新城区。

　　2004年是实现五年奋斗目标和整体要求的关键一年。今年全市经济社会发展的主要预期目标是：全市生产总值增长12%左右，全社会固定资产投资增长12%，社会消费品零售总额增长12%，外贸出口增长6%，实际吸收外商直接投资增长12%，地方财政一般预算收入增长10%，居民消费价格上升1%左右，城镇登记失业率控制在3.2%左右，人口自然增长率控制在8‰以内，在岗职工平均工资增长7.5%，农村居民人均纯收入增长4%。

为实现上述目标，必须做好以下工作：

　　一、突出工业主导地位，加快工业结构调整优化升级

　　推进工业重点项目建设。在抓好中小企业发展的基础上，把培育和引进大企业、大项目作为兴工富市的关键来抓。重点推进阳江核电站、阳西火力发电厂、阳江抽水蓄能电站、海陵岛风力发电场等项目的前期工作和建设，打造广东电力能源基地。大力发展与电力相关产业，带动工业跨越式发展。加快发展临海工业，重点推进丰源粮油粮食加工城建设，做好临海石油、化工及物流配送的规划工作。抓好县域重点工业项目的建设，加快县域工业经济的发展。

改造提升支柱产业。认真转化和应用省工业产业竞争力研究成果，走新型工业化道路。抓好五金刀剪、食品加工、建材机电、纺织服装、医药化工、家具编织等传统产业的优化升级。鼓励和支持企业增加技改投入，推进阳东县纳谷五金电器有限公司、阳春市兴利工艺厂等一批重点工业企业的技术改造。大力实施技术创新工程，建立以企业为主体的技术创新体系。加快实施名牌带动战略，争创6个国家级和省级名牌。

　　做大做强龙头企业。开展评选阳江市“工业企业20强”活动。大力扶持喜之郎、谊林海达、宝马利汽车空调等一批带动能力强的龙头企业，优化资源配置，实现规模扩张。继续支持阳江十八子集团、广东宏鹰集团、阳西永光集团等五金刀剪企业进一步做大做强，带动行业加快发展。

　　发展高新技术产业。加大高新区基础设施建设力度，高起点规划，高标准建设，高效能管理，努力把高新区和各类园区办成阳江发展新型工业的重要基地，加大招商引资力度，争取在发展汽车配件、电子信息、生物工程、医药、新材料和环保等高新技术产业取得突破，促进工业结构优化升级，加快工业化进程。

　　二、大力发展农业和农村经济，促进城乡协调发展

　　以增加农民收入为核心发展农业农村经济。全面贯彻今年中央1号文件精神，把促进农民增收作为“三农”工作的重中之重。加大农业结构调整力度，在切实保护耕地和提高粮食综合生产能力，确保粮食安全的前提下，进一步调整粮经比例，大力发展特色水果、蔬菜、南药等作物。加快发展品牌农业，突出抓好“一镇一业”、“一村一品”，促进特色农业和优势农产品产业带的形成，提高农产品的竞争力和经济效益。加强农业科研和推广网络建设，推广良种良法，推行农业标准化生产，发展优质、高效、高产、生态、安全农业。加快推进农业产业化经营，培育发展农业龙头企业，增强带动农户发展商品经济能力。大力发展农产品加工、流通业，建设完善农产品十大专业批发市场。加强农业信息网络建设，切实为农民提供市场信息和产前、产中、产后等社会化服务。突出抓好以农田水利、县镇村公路为重点的基础设施建设，增强农业发展后劲。加快农村富余劳动力转移，搞好劳务输出，拓宽农民增收渠道。加快林业发展，不断提高生态林功能等级和商品林经济效益。积极发展畜牧业，进一步提高畜牧业在农业中的比重。切实抓好7个国家级、9个省级、3个市级农业示范区建设，推进农业机械化，不断提高农业现代化水平。

　　全面推进城镇化进程。制订实施城镇化发展纲要，完善城乡规划体系和城乡规划实施监控体系，形成政府引导、科学规划、市场运作、产业支撑的城镇化机制。以建设中心镇为突破口，以户籍制度改革为重点，以产业发展为依托，创新城乡管理体制，引导和鼓励各种生产要素加快向县城和中心镇集聚，增强它们吸纳农村人口、带动农村发展的能力。

切实减轻农民负担。抓好农村土地经营体制改革，逐步建立土地使用权有偿转让的流转机制。巩固农村税费改革成果，按照中央和省的要求，逐步取消农业税。整顿清理涉农行政事业性和经营服务性项目收费，完善涉农收费公示制。

　　三、积极发展特色产业，壮大区域特色经济

　　大力推进民营经济发展提高。发展民营经济要在“放、调、扶、新、优”等下功夫。“放”就是进一步放宽政策，努力消除民营经济发展的体制性和政策性障碍，真正为民营企业营造“政治平等、政策公开、法律保障、放手发展”的良好环境。“调”就是调整结构，优化升级，引导民营企业大力发展传统产业、特色产业，发展科技型、外向型、农产品加工型、就业型和扶贫型企业，鼓励民间资本投向基础设施建设、旅游开发、现代服务业和高新技术产业，优化民营经济的产业结构。“扶”就是加大扶持力度，进一步落实省、市《关于加快民营经济发展的决定》和12个配套文件，重点解决好贷款难问题。“新”就是引导民营企业创新体制，创新技术，创新管理，力促民营企业现代化。“优”就是进一步优化服务，努力为民营企业提供全方位、全程式的服务。

　　努力做强海洋产业。以加快发展海洋渔业为突破口，以建设海洋强市为目标，通过抓龙头、抓品牌、抓基础、抓提升，提高海洋经济综合素质，做强海洋产业。以水产品加工和流通为龙头，以科技兴渔为手段，带动远洋捕捞和海水养殖，促进渔工贸一体化，发展渔业产业化经营，提高渔业效益。下大力气打造程村蚝、东平鱼翅、“南海开渔节”和国家中心渔港等品牌。推进现代渔港经济区建设。积极发展临海工业、滨海旅游业和海洋运输业，全面发展海洋经济。

　　着力做大旅游产业。实施旅游发展总体规划，加快重点景区和旅游线路建设。进一步整合资源优势，挖掘文化内涵，发展旅游商品。培育旅游热线，打造滨海生态休闲游、岩洞观光游、温泉健身游、传统工业特色游等区域特色旅游品牌。加大旅游推介促销力度，办好第五届阳江旅游文化节。充分发挥市旅游协会的作用，切实加强旅游行业自律和管理，提高服务水平，营造发展旅游的大环境。

　　四、大力推进基础设施和城市建设，积极有效扩大内需

　　抓好基础设施和城市建设，扩大投资规模。加大电网建设力度，切实解决电网“卡脖子”问题。继续配合省加快阳茂高速公路阳江段的施工，确保年底前建成通车。积极推进省道S113线阳春南段一级公路、S369线春湾至罗定二级公路、S282线新墟至月亮湾二级公路和那龙到新洲的公路改建。进一步完善阳江港的规划，加快建设两个在建的3万吨级和3.5万吨级码头。抓好市区、阳春、阳西的城市防洪工程建设，着力解决连环、上水、东湖、合水等四个水库和阳东台平三丫、江城四、海陵新盐、阳西白土等联围的除险加固和达标建设。改造东风四路和建设石湾北路、体育北路、高凉路、河堤南路、鸳鸯湖外环湖路，加快文化广场、世纪公园等工程施工进度，做好人防设施、无障碍设施和公共停车场等的规划建设，推进一批环境优美的花园式住宅小区和“一江两岸”的开发。积极开展创建“国家卫生城市”活动，进一步搞好城市净化、绿化、亮化、美化工作，使城市环境卫生有根本性的好转。

　　多种形式开展经贸合作，积极有效拓展国内市场。坚持市场开拓与经贸合作相结合，积极组织企业参加省举办的首届“泛珠三角”区域产品展示暨经济技术合作洽谈会、第三届珠江三角洲地区与山区经济技术合作洽谈会及其它经贸活动，大力推进与省内外的经贸合作，开拓国内市场。以举办第三届刀剪博览会和第五届旅游文化节等重大活动为契机，开展各种经贸活动，提高经贸合作层次，提高“阳货”的国内市场占有率。

　　大力发展第三产业，培育新的消费热点。继续发展商业零售、餐饮娱乐等传统服务业，积极发展连锁经营、物流配送、电子商务等流通方式，加快发展现代物流业。大力发展保险、会展等新兴服务业和中介服务。培育和扩大住房、汽车、电信、教育、文化、医疗、体育健身和旅游休闲等消费热点，增加即期消费，推动消费结构升级，增强消费对经济的拉动作用。

　　五、扩大对外开放，加快发展外源型经济

　　努力扩大外贸出口。充分发挥粤港澳实施CEPA的优势，努力做好外经贸工作。继续实施科技兴贸，优化出口产品结构，提高出口效益和竞争力。推动出口市场多元化，拓展新的市场空间，巩固提高美、日、欧和港澳等传统出口市场，积极开拓俄罗斯、东欧、拉美、非洲等新兴市场。创建“中国五金制品（阳江刀剪工具）出口基地”，办好第三届中国（阳江）国际刀剪博览会，帮促五金刀剪行业开拓国际市场。深化外经贸体制改革，支持民营企业扩大出口，推进外贸主体多元化。加强口岸建设和管理，营造良好的口岸通关环境。

　　提高招商引资水平。坚持环境是发展第一要素，把“亲商、安商、富商”作为第一投资环境来抓，着力打造“诚信阳江”和“开放阳江”，全方位构筑有利于扩大对外开放和招商引资的服务平台。完善招商引资工作机制，实行招商引资目标管理和奖惩制度，充分调动全民招商积极性。整合资源，办好市县工业园区，坚持主动“走出去”和积极“请进来”相结合，积极开展项目招商、产业招商、园区招商、网上招商、委托招商。加大力度吸引美日欧等国家和地区的投资，吸引跨国公司来我市设立研发机构、制造基地。进一步密切与港澳台的经贸联系与合作，更加积极主动地承接发达地区的产业转移。推进与“大珠三角”和周边城市的优势互补，促进在工业、农业、服务业、物流业和旅游业等的交流与合作。

　　六、做好就业和社保工作，改善人民生活

　　全面推动就业和再就业工作。坚持劳动者自主择业、市场调节就业和政府促进就业的方针，推进城乡统筹就业。坚持用发展的办法促进就业和再就业，实施积极的就业政策，大力发展民营经济、劳动密集型企业以及旅游、商贸、饮食和社区服务等第三产业，扩大就业门路，增加就业岗位。以市劳动力中心市场和技工学校为龙头，加快公共就业服务平台建设，加强下岗失业人员再就业培训，帮助失业人员特别是“4050”人员的再就业。加强农村富余劳动力转移就业培训，不断提高劳动者创业和就业能力。重视做好高校毕业生就业工作。加强劳动监察，切实保障劳动者合法权益。

　　大力推进和完善社会保障体系建设。抓好个体私营等非公有制企业和灵活就业人员的参保工作，扩大社会保险覆盖面。完善企业职工基本养老保险制度，坚持社会统筹与个人账户相结合，逐步做实个人账户。大力推进城镇职工医疗保险，完善工伤、生育保险制度。加大社保金征缴力度，健全社保调剂金制度，加快推进养老保险市级统筹。全面做好“两个确保”和“三条保障线”工作，完善城乡低保制度，扩大农村低保范围。

切实解决群众生产生活困难。加快实施“十项民心工程”。继续推进农村贫困户泥砖住房改造，改善农民居住条件。完善扶贫开发机制，抓好农村扶贫工作，千方百计增加农民收入。关心城乡低收入群众，重点解决困难群众住房难、子女入学难、就医难、打官司难等问题。

　　七、深化各项改革，完善社会主义市场经济体制

　　大力推进国有企业改革。以深化国有企业产权制度改革为重点，加快国有企业的公司制、股份制改造，加快建立现代企业制度。积极稳妥地推进劣势企业退出市场，促进国有资本向优势产业集聚，放开搞活国有中小型企业。积极引导和鼓励民营企业参与国有企业的改组改造，大力发展混合型所有制经济。加快垄断行业改革，放宽市场准入限制，引入竞争机制，逐步改变垄断行业产权单一的状况。加强国有资产的管理、监督和运营，确保国有资产的保值增值。

积极推进投资体制改革。按照国家投资体制改革方案精神，认真落实“谁投资、谁决策、谁收益、谁承担风险”的要求，扩大企业投资管理自主权。对国家没有明令禁止的项目，都向民资、外资开放，鼓励他们参与投资建设。全面推行社会投资基本建设项目登记备案制度，对企业不使用政府性资金投资建设的重大项目及非限制类项目，政府主要从维护经济安全、合理开发利用资源、保护生态环境、优化产业布局、保障公共利益、防止垄断等方面进行审批或核准，逐步建立市场引导投资、企业自主决策、银行独立审贷、融资方式多样、中介服务规范、宏观调控有效的新型投资体制。

　　继续整顿和规范市场经济秩序。进一步规范市场主体和市场行为，完善有形建筑、土地交易和产权交易市场运作机制。规范中介机构活动。严厉打击传销和变相传销、制假售假、走私贩私、偷税逃税以及侵犯知识产权行为，维护市场经济秩序的稳定。强化市场物价监管，防止无序与恶性竞争。加强政府、企业和社会信用建设，维护本地区良好信用形象。

　　切实加强安全生产工作。认真落实《安全生产法》，加大安全执法力度，加强安全生产基础工作，强化安全生产监管，推行注册安全主任制度，建立安全生产长效机制，严防重特大安全生产事故发生。加强食品药品安全监督。重视社会安全工作。

　　八、加强财税金融工作，为经济社会发展提供财力和金融支持

　　严格依法治税，强化税收征管。推进私营企业、个体工商户建账制度，在加强主体税种征管的同时，切实抓好非主体税种、国有资产经营收益及其他非税收入的征管工作，做到应收尽收。加大税务稽查力度，大力清缴企业欠税，严厉打击各种偷、逃、骗、抗税行为，确保应收税款及时足额收缴入库。

　　加强财政管理，严肃财经纪律。严格预算管理，按照保工资、保社保、保教育、保稳定、促发展的顺序和要求，调整优化支出结构，严格控制和压缩一般性支出，确保公共事业性经费的支出，提高财政运作水平。完善“收支两条线”制度，加强预算外资金管理。完善委派财务总监制度，加强审计工作，加大对政府工程的监督力度，强化政府性基金、财政专项资金和财政支付转移资金的监管，严厉查处各种违反财经纪律的行为。

　　深化财政改革，完善公共财政体制。积极探索财政投融资体制改革，对确需财政投入的建设资金，改无偿拨款为有偿使用。建立财政抗风险应急预警机制，提高防范财政风险的能力。建立财政支出绩效评价体系，并将财政支出绩效评价结果作为今后预算安排的重要依据。

　　强化金融资产监管。进一步加大城信社和农金会的资产清收力度，继续做好防范金融风险工作。积极推进农村信用社体制改革，加强金融监管，维护金融秩序稳定。树立现代银行营销理念，优化和配置资源，大力扶持民营、海洋、旅游经济以及中小企业的发展。加快建立中小企业信用担保体系，切实解决企业贷款难与银行难贷款的矛盾，实现银企互动互惠。

　　九、加强环境保护和生态建设，增强可持续发展能力

　　重视环境保护和综合治理。积极创建“国家环保模范城市”，全面实施治污保洁工程。进一步扩大市区污水处理能力，积极推进阳东、阳春、阳西、海陵污水处理厂的建设。加快市区奕垌垃圾填埋场的改造和生活垃圾渗滤液处理厂、市医疗废物处理中心的建设，减少城市垃圾和医疗废物的污染。加强对机动车辆尾气污染治理和建筑垃圾的管理。

努力提高水环境质量。认真实施《漠阳江水质保护规划》，全面整治漠阳江两岸的污染源，重点加强春城河段和江城河段的监测和治理。建设漠阳江水质自动监测站，控制污染物排放总量，切实保护漠阳江水资源。加快漠阳江千里翠竹长廊生态工程的可行性研究，力促早日实施。

　　进一步抓好生态建设。开展创建“国家园林城市”活动，大力推进林业生态县建设。重视湿地、红树林资源和鸳鸯湖的保护，抓好自然保护区、森林公园的建设。建设好水源涵养林、沿海防护林、农田林网以及公路两侧的绿色通道。抓好海洋自然保护区和人工鱼礁的建设，切实保护海岸带和海洋环境。继续开展创建生态示范村(镇、场、园)活动，大力推进农村环境整治工作，切实加强农田环境保护。树立新的城市发展观，建设生态文明城市。

　　提高人口与计划生育工作水平。进一步贯彻落实《人口与计划生育法》、《广东省人口与计划生育条例》等法律法规，推进人口与计划生育工作全面进入法制轨道。继续加强计划生育基层基础建设，真正把计划生育工作的重心下移到镇和村。加大综合治理出生人口性别比的力度，加强流动人口计划生育管理，大力开展计划生育优质服务，完善计划生育村（居）民自治和利益导向机制。加快推进计划生育综合改革和信息化建设，提高计划生育管理和服务水平。

　　十、推动文化名城建设，促进经济社会协调发展

　　加快教育改革与发展。继续把教育摆上优先发展位置，加快教育管理体制、办学体制和教育理念的创新。切实落实“以县为主”的管理体制，全面实行县级党政主要领导基础教育工作考核督导制度。加大教育投入，鼓励支持和规范多种形式的社会办学。整合教育资源，调整教育布局，优化教育结构，改造薄弱学校，加强农村教育工作。加快发展幼儿教育，巩固提高九年义务教育，大力发展高中阶段教育和职业技术、技工教育，办好高等教育。推进学校人事制度改革，加强校长和教师队伍建设。加快教育信息化进程，推行课程综合改革，全面实施素质教育，提高教育质量。积极推进阳江一中、阳春一中创建全国示范性普通高中。巩固提高成人教育，加强继续教育，加快构建终身教育体系。

　　大力推进科技创新。制订科技发展中长期规划，继续实施“阳江市科技创新9项计划”，滚动实施“双十工程”。办好现有20家省、市级工程技术研发中心，择优扶持年产值超5000万元的工业企业组建工程技术研发中心，加快建立以企业为主体的技术创新体系。认真抓好平冈镇、东城镇两个省级专业镇技术创新工作，启动市级专业镇技术创新试点。加强科技服务中介机构建设。做好知识产权保护和专利申请工作。重视科普工作。

　　加强文化名城建设。以 “南海1号”沉船打捞和建设广东海上丝绸之路博物馆为契机，发展具有地方特色和优势的文化产业，培育新的经济增长点。发挥“南国风筝之乡”优势，培育阳江风筝文化品牌。推进具有阳江特色的商品品牌文化、旅游文化以及传媒、演艺、体育、会展、出版等优势文化的产业化。积极挖掘、保护民俗文化遗产，做好文物古迹的保护和开发工作。加强文化基础设施建设，加快市文化广场、图书馆、科技馆、博物馆、群众艺术馆的建设。

　　实施人才强市战略。树立人才资源是第一资源观念。创新人才引进政策，大力招才引智，积极引进各类高层次人才到阳江创业。大力推动市技工学校创建国家级技工学校，加快各类高素质技术人才的培养。健全各类人才的选拔任用、考核评价、激励监督机制，促进优秀人才成长。办好落户阳江的粤西区域性人才市场，发挥人才资源市场化配置的作用。

　　十一、加强社会主义精神文明和民主法制建设，发展社会各项事业

　　开展形式多样的精神文明创建活动。认真贯彻《公民道德建设实施纲要》，开展社会公德、职业道德和家庭美德教育，加强以诚信为重点的公民道德建设，弘扬务实进取、开放兼容、敬业奉献的精神，营造团结和谐的人文环境。继续开展多种形式的精神文明创建活动，提高城市文明程度。

　　加强社会主义民主法制建设。继续加强和完善重大问题集体决策制度、专家咨询制度、社会公示和社会听证制度以及决策责任制度，提高科学民主决策水平。坚持政府向人大及其常委会报告工作制度，积极支持人民政协履行政治协商、民主监督、参政议政职能，认真听取民主党派、工商联、无党派人士及各人民团体的意见建议。抓好以《中华人民共和国宪法》为核心的“四五”普法教育，做好《中华人民共和国行政许可法》的学习、宣传和贯彻实施工作，提高公职人员依法办事意识，增强公民的法律素质。加强政府法制建设，完善政务公开制度，强化对行政许可的监督，规范行政执法行为，营造良好的法制环境，全面推进依法治市。严密防范和打击“法轮功”等邪教组织的各种破坏活动。大力开展“严打”整治斗争，严厉打击各种刑事犯罪活动。继续抓好“禁毒03工程”，铲除黄赌毒等社会丑恶现象。全面推进社区警务建设，加强城乡社会治安综合治理，下大力气整治市区治安。重视做好群众来信来访工作，继续落实领导干部接访日制度，办好“市长热线”电话，妥善处理人民内部矛盾。

　　发展卫生、体育等各项社会事业。继续抓好疾病预防控制、卫生监督执法、医疗救治三大体系建设，建立应对突发性公共卫生事件长效机制。大力推进“全民安康工程”。重视做好非典、禽流感以及艾滋病等传染病的防治工作。加快中医药事业发展。完善药品集中采购制度。继续深化医疗卫生体制改革，加快办医多元化步伐，鼓励民资、外资参与医疗机构改造。抓好乡镇薄弱医院改造，优化组合农村医疗网点。建立完善新型农村合作医疗制度，提高农村合作医疗人口覆盖率。深入开展爱国卫生活动，提高农村自来水和卫生厕所普及率。加快公共体育场馆和设施建设步伐，完善全民健身广场各项配套设施建设，广泛开展全民健身活动。认真备战第十二届省运会，办好首届世界华人风筝锦标赛、全国男子柔道冠军赛和市第五届体育节。加强国防教育，做好“拥军优属、拥政爱民”和民兵预备役工作，不断提高双拥共建活动水平。加强人防工作，逐步完善防震减灾措施。全面落实宗教、民族政策，依法管理宗教事务，进一步做好民族工作。重视做好老龄工作。切实保护妇女儿童合法权益，加快发展妇女儿童事业。继续重视和做好外事、侨务、台务、统计、民政、档案、市志等工作。

　　加强机关作风建设和廉政建设。加快市级政府机构职能调整，切实改进政府管理经济的方式，逐步实现主要运用经济和法律手段管理经济，提高政府管理经济社会的水平。加快电子信息网络建设，推进电子政务。加强《中国共产党党内监督条例（试行）》、《中国共产党纪律处分条例》的学习，坚持“两个务必”，努力做到“五个力戒”，干净干事，切实加强勤政廉政建设。坚持不懈地开展反腐败斗争，继续开展行风检查和评议工作，坚决纠正损害群众利益的不正之风。强化政府机关及其工作人员的行政监察。进一步改进机关工作作风，弘扬求真务实精神，大兴调查研究之风，努力为基层和群众多办好事实事。今年继续为群众办好10件实事：（1）抓好老区、山区37所危房学校改造；（2）抓好白沙、石湾、永宁3个110KV输变电工程及农城网工程改造建设；（3）建设500公里镇通村公路硬底化工程；（4）实施市教育信息化工程，实现中心小学电脑室建设；（5）创建广东省卫生镇、卫生村；（6）建设市人民医院感染性疾病中心大楼；（7）逐步建立和完善市区治安防控体系；（8）建设150套经济适用住房和廉租住房；（9）抓好宁正路、正坑路、平湾路、豪贤路等4条道路的路灯建设；（10）建设华南理工大学阳江五金刀具研究院。

　　各位代表！回顾过去，我们豪情满怀。展望未来，我们既信心百倍，又深感任重道远。让我们紧密团结在以胡锦涛同志为总书记的党中央周围，以邓小平理论和“三个代表”重要思想为指导，认真贯彻落实省委、省政府和市委的各项决策，解放思想，实事求是，与时俱进，开拓创新，为我市加快发展、跨越发展、协调发展，实现与全省同步进入全面小康社会而努力奋斗！
